# Serous retinal detachment secondary to bilateral choroidal osteoma successfully treated with subscleral sclerectomy: A case report

**DOI:** 10.1016/j.ajoc.2025.102249

**Published:** 2025-01-07

**Authors:** Satoko Fujimoto, Kazuichi Maruyama, Takuya Shunto, Kohji Nishida

**Affiliations:** a Department of Ophthalmology, Osaka University Graduate School of Medicine, 2-2 Yamadaoka, Suita, Osaka, 565-0871, Japan; b Department of Vision Informatics, Osaka University Graduate School of Medicine, 2-2 Yamadaoka, Suita, Osaka, 565-0871, Japan; c Integrated Frontier Research for Medical Science Division, Institute for Open and Transdisciplinary Research Initiatives, Osaka University, 2-2 Yamadaoka, Suita, Osaka, 565-0871, Japan

**Keywords:** Choroidal osteoma, Serous retinal detachment, Subretinal fluid, Sclerectomy, Subscleral sclerectomy, Scleral window

## Abstract

**Purpose:**

To report a case of bilateral choroidal osteoma successfully treated with subscleral sclerectomy for secondary serous retinal detachment (SRD).

**Observations:**

A 52-year-old Japanese woman first diagnosed with Vogt-Koyanagi-Harada disease and treated with steroids for 9 years was referred to our clinic. SRD in both eyes recurred frequently and was uncontrolled with adalimumab subcutaneous injections and oral cyclosporine, in addition to steroids. A yellowish-to-orange, slightly elevated subretinal lesion was observed superior to the macular and inferotemporal regions of the right eye and superior to the macular and temporal regions of the left eye without any inflammation. Swept-source optical coherence tomography (OCT) revealed SRD in the fovea and a mass under the retinal pigment epithelium (RPE) in the macular area of both eyes. Indocyanine green angiography (ICGA) demonstrated hypocyanescence corresponding to the mass area under the RPE, with dilation of the dominant vortex veins. Ultrasonography revealed a hyperechogenic mass in the posterior wall of both eyes with deeper acoustic shadows, and computed tomography (CT) detected calcifications in the posterior wall of both eyes. A bilateral choroidal osteoma was diagnosed, and the superior SRD of her left eye increased toward the fovea without any evidence of choroidal neovascularization during follow-up. Therefore, subscleral sclerectomy (4 × 4 mm^2^ sclerectomy under the scleral flap) was performed at three sites at the equators in the upper temporal, upper nasal, and lower temporal quadrants of her left eye. Immediately after surgery, SRD resolved dramatically. As the foveal SRD of her right eye also increased after two months, the same surgery was performed, and it worked successfully.

**Conclusions and Importance:**

Choroidal osteoma can cause severe SRD that cannot be controlled with medication. Although further studies are needed, subscleral sclerectomy may be an effective treatment for the resolution of subretinal fluid secondary to choroidal osteoma by improving choroidal circulation congestion.

## 1 Introduction

A choroidal osteoma is a benign ossifying tumor within the choroid with multiple short-branching tufts of vessels on the tumor surface. Patients with choroidal osteoma are commonly asymptomatic and have well-preserved visual acuity; however, they may be treated for secondary complications, including disruption of the retinal pigment epithelium (RPE), atrophy of photoreceptors, subretinal fluid (SRF), and choroidal neovascularization (CNV). In particular, treatment for SRF, such as anti-vascular endothelial growth factor (VEGF) injections, laser photocoagulation, photodynamic therapy (PDT), or transpupillary thermotherapy (TTT).^1^, ^2^, ^3^, ^4^. In this case report, subscleral sclerectomy successfully resolved serous retinal detachment (SRD) caused by bilateral choroidal osteoma.

## 2 Case report

A 52-year-old Japanese woman first diagnosed with Vogt-Koyanagi-Harada (VKH) disease and treated with intravenous, oral, sub-tenon, and topical steroids, but with frequent recurrence of SRF for 9 years, was referred to our clinic. SRF in both eyes recurred and was uncontrolled even after adalimumab subcutaneous injections and 3–4 mg/kg oral cyclosporine in addition to steroids. Ophthalmic examination revealed a best-corrected visual acuity of 20/25 in the right eye and 20/20 in the left eye, with a slightly higher intraocular pressure (IOP) (right, 20 mmHg; left, 21 mmHg) without any inflammation.

**Fig. 1:**
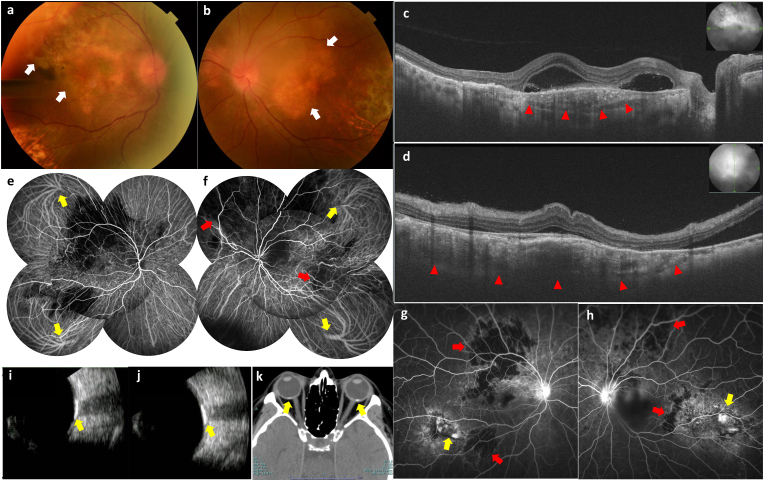
Color photographs, posterior segment optical coherence tomography (PS-OCT) images, indocyanine green angiography (ICGA) images, fluorescence angiography (FA) images, Ultrasonographic images, and computed tomography (CT) images of bilateral choroidal osteoma. **(a, b)** Color photographs of the right eye **(a)** and the left eye **(b)** showing the yellowish to orange, slightly elevated subretinal lesion (black arrows). **(c)** Horizontal PS-OCT images of the right eye with subretinal fluid and a mass under retinal pigment epithelium (RPE) (red arrowheads). **(d)** Vertical PS-OCT images of the left eye with subretinal fluid and a mass under RPE (red arrowheads). **(e, f)** Early-phase ICGA images of the right eye **(e)** and the left eye **(f)** showing partial hypocyanine choroidal circulation (red arrows) and dilated choroidal vortex veins (yellow arrows). **(g, h)** intermediate-phase FA images of the right eye **(g)** and the left eye **(h)** showing hypofluorescent spots (red arrows) and hyperfluorescent spots (yellow arrows). **(i, j)** Ultrasonographic images of the right eye **(i)** and the left eye **(j)** showing hyperechogenic mass at the posterior wall of both eyes with deeper acoustic shadows (yellow arrows). **(k)** CT showing calcification of the choroid at the posterior wall of both eyes (yellow arrows). (For interpretation of the references to colour in this figure legend, the reader is referred to the Web version of this article.)

**Fig. 2:**
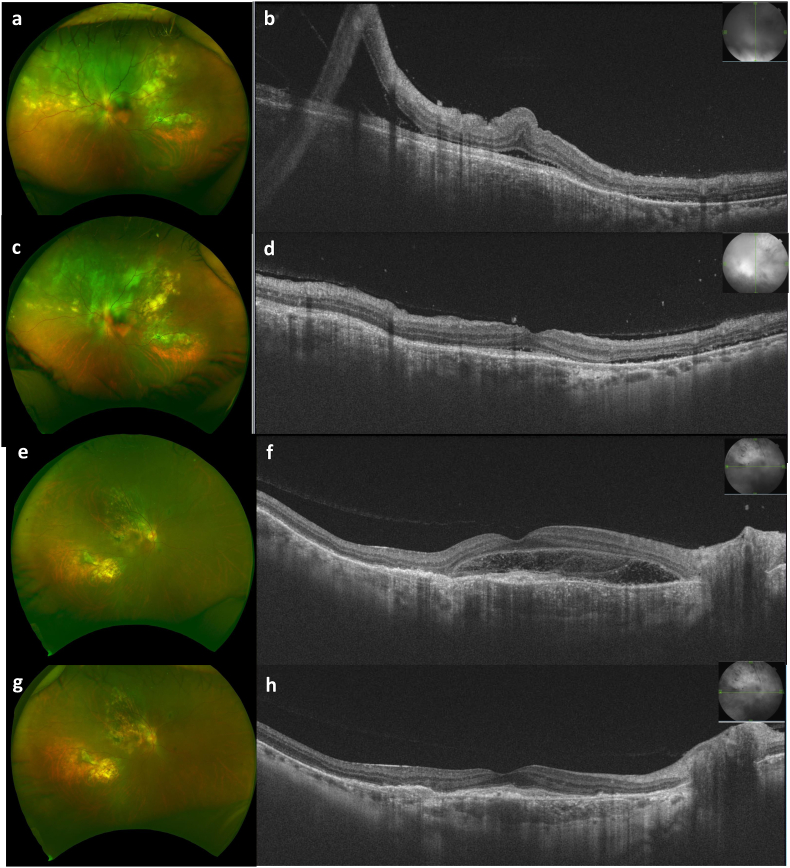
Color photographs and posterior segment optical coherence tomography (PS-OCT) images before and after the subscleral sclerectomy for secondary serous retinal detachment (SRD). **(a,b)** Color photographs of the left eye before **(a)** and after **(c)** the surgery. **(c,d)** Vertical PS-OCT images of the left eye before **(b)** and after **(d)** the surgery. Superior SRD resolved after the surgery. **(e,f)** Color photographs of the right eye before **(e)** and after **(g)** the surgery. **(f,h)** Horizontal PS-OCT images of the left eye before **(a)** and after **(b)** the surgery. SRD at the fovea resolved after the surgery. (For interpretation of the references to colour in this figure legend, the reader is referred to the Web version of this article.)

In the fundus, yellowish-to-orange, slightly elevated subretinal lesions were detected superior to the macular and inferotemporal regions of the right eye, and superior to the macular and temporal regions of the left eye. (Fig. 1a and b). Swept-source optical coherence tomography (OCT) (DRI-OCT, Topcon, Tokyo, Japan) revealed a slight SRF in the fovea and a mass under the RPE in the macular area of both eyes (Fig. 1c and d). Indocyanine green angiography (ICGA) demonstrated hypocyanescence superior to the macular and inferior regions of the right eye and superior to the macular and temporal regions of the left eye from the early phase, corresponding close to the mass area under the RPE in OCT and the yellowish-to-orange lesions in the fundus photographs (Fig. 2e and f). Additionally, the upper and lower temporal vortex veins were dilated in both eyes, whereas the upper and lower nasal vortex veins were not. Fluorescein angiography (FA) revealed hypofluorescent spots under intact retinal vascular perfusion superior to the macular region of the right eye, superior to the macular temporal region of the left eye, and hyperfluorescent window defects in the inferotemporal and temporal regions of the right and left eyes, respectively (Fig. 1g and h). Ultrasonography detected a hyperechogenic mass in the posterior wall of both eyes with deeper acoustic shadows (Fig. 1i and j), and computed tomography (CT) detected calcification of the choroid in the posterior wall of both eyes (Fig. 1k). There were no complaints other than the eyes, and no abnormalities were found in chest X-rays, electrocardiograms, blood tests, and urine tests.

**Fig. 3:**
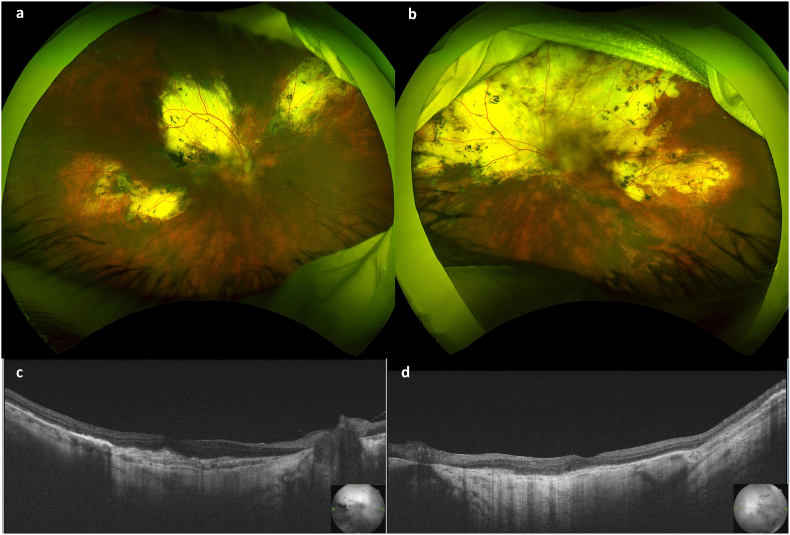
Color photographs and posterior segment optical coherence tomography (PS-OCT) images four years after the subscleral sclerectomy. **(a,b)** Color photographs of the right eye **(a)** and the left eye **(b)**. **(c,d)** Horizontal PS-OCT images of the right eye **(c)** and the left eye **(d)**. (For interpretation of the references to colour in this figure legend, the reader is referred to the Web version of this article.)

The patient was diagnosed with bilateral choroidal osteoma without inflammation. Under 7.5 mg/week, oral methotrexate was administered with 0.5 mg/kg of oral steroid and sub-tenon triamcinolone acetonide to check whether SRF decreased through immunosuppression. The institutional review board of Osaka University Hospital approved the prescription of oral methotrexate for refractory uveitis with written informed consent (Work Order #03-M022). However, the SRF did not decrease but rather increased superior to the macula without any retinal holes or tears in the left eye (Fig. 2a and b), and vision declined to 20/60 in 4 months. Therefore, a subscleral sclerectomy (4 × 4 mm^2^ sclerectomy under the scleral flap) was performed at three sites at the equators in the upper temporal, upper nasal, and lower temporal quadrants. One day after surgery, the SRF resolved dramatically (Fig. 2c and d). After two months, the SRF at the fovea increased and vision declined to 20/50 in the right eye (Fig. 2e and f), and subscleral sclerectomy was performed. Four days after surgery, the SRF resolved (Fig. 2g and h). After surgery, vision was 20/25 in the right eye and 20/25 in the left eye with the cataract. After 6 months, a new choroidal osteoma and SRF were detected supernasally in the left eye with a higher anterior flare, which resolved gradually with 200 mg/day of non-steroidal anti-inflammatory drugs (NSAIDs) and 7.5 mg/week of oral methotrexate. Oral steroids were gradually reduced and completely discontinued 2 years after surgery. CNV occurred and was treated with an anti-VEGF injection at the fovea in the left eye four months after the steroid was discontinued. Overall, inflammation was controlled under 7.5 mg/week of oral methotrexate and NSAIDs for 4 years of follow-up with a vision of 20/80 and 20/50 under the cataract (Fig. 3a–d).

## 3 Discussion

We describe a case of bilateral choroidal osteoma that was treated successfully with subscleral sclerectomy to resolve SRF. Subscleral sclerectomy is effective in primary uveal effusion syndrome with a firm, abnormal sclera, congestion of the vortex vein, and SRF accumulation from dysfunction in the pump mechanism of the RPE,^5^, ^6^, ^7^ by reduction resistance to transscleral fluid outflow. We demonstrated a successful subscleral sclerectomy that resolved SRF caused by bilateral choroidal osteoma. NSAIDs, oral methotrexate, and anti-VEGF injections were administered after subscleral sclerectomy, but only when a new choroidal osteoma or CNV occurred.

In the present case, the patient was diagnosed with VKH before presentation. Intraocular inflammation causes dystrophic intraocular calcification and ossification,^8^ and Ozawa et al. reported a case of bilateral choroidal osteoma after intraocular inflammation caused by VKH,^9^ similar to our case. Before surgery, oral methotrexate, which reduces severe inflammation in refractory uveitis,^10^, ^11^, ^12^ did not resolve the SRF in this case; however, after surgery, oral steroids were tapered off with oral methotrexate and NSAIDs. We speculate that uveitis and choroidal osteoma coexisted in this case, and anti-inflammatory medication should have been continued to control the uveitis. Histiocytosis could be considered as a differential diagnosis. Although a PET scan was not performed in this case, if histiocytosis is diagnosed, treatment with targeted agents, particularly those targeting the MAP kinase pathway, could be considered.

Reportedly, SRF not associated with CNV in choroidal osteoma may arise from pinpoint leakage sites over the tumor^13^ or accumulate through a degenerated outer blood-retinal barrier due to gradual atrophy of the overlying RPE and Bruch membrane,^14^ and are treated differently, such as anti-VEGF injections, laser photocoagulation, PDT, or TTT.^1^, ^2^, ^3^, ^4^. We did not attempt laser photocoagulation to aid in the subretinal fluid resolution in this case because laser treatment itself sometimes induces severe inflammation in eyes with uveitis, especially when inflammation is not being controlled. Because ICG revealed congestion of the choriocapillaris perfusion by hypocyanescence at the sites of choroidal osteoma and dilation of the dominant vortex veins, the success of subscleral sclerectomy in this case may indicate that it improved the congestion of choroidal circulation and may be an effective treatment option. However, further studies are needed to confirm whether subscleral sclerectomy is useful for secondary SRF caused by choroidal osteoma.

## 4 Conclusion

In summary, we report a case of bilateral choroidal osteoma that was successfully treated with subscleral sclerectomy for secondary SRD. Choroidal osteomas sometimes cause severe SRD that cannot be controlled with medication. Although further studies are required, subscleral sclerectomy may be effective in improving choroidal circulation congestion.

## CRediT authorship contribution statement

**Satoko Fujimoto:** Writing – original draft, Data curation. **Kazuichi Maruyama:** Writing – review & editing, Methodology, Investigation, Conceptualization. **Takuya Shunto:** Writing – review & editing. **Kohji Nishida:** Supervision.

## Patient consent

The patient provided written consent for the publication of medical details and photographs.

## Authorship

All authors attest that they meet the current ICMJE criteria for authorship.

## Funding

This study did not receive any funding or grants.

## Declaration of competing interest

The authors declare that they have no known competing financial interests or personal relationships that could have appeared to influence the work reported in this paper.
